# Novel, Broadly Reactive Anticapsular Antibodies against Carbapenem-Resistant Klebsiella pneumoniae Protect from Infection

**DOI:** 10.1128/mBio.00091-18

**Published:** 2018-04-03

**Authors:** Elizabeth Diago-Navarro, Michael P. Motley, Gonzalo Ruiz-Peréz, Winnie Yu, Julianne Austin, Bruna M. S. Seco, Guozhi Xiao, Aniska Chikhalya, Peter H. Seeberger, Bettina C. Fries

**Affiliations:** a Department of Medicine, Infectious Disease Division, Stony Brook University, Stony Brook, New York, USA; b Department of Molecular Genetics and Microbiology, Stony Brook University, Stony Brook, New York, USA; c Universidad Francisco de Vitoria, Pozuelo de Alarcón, Madrid, Spain; d Department of Biomolecular Systems, Max Planck Institute of Colloids and Interfaces, Potsdam, Germany; Louis Stokes Veterans Affairs Medical Center

**Keywords:** adjuvant therapy, antibiotic resistance, carbapenem resistance, infection protection, *Klebsiella pneumoniae*, monoclonal antibodies

## Abstract

Carbapenem-resistant (CR) sequence type 258 (ST258) Klebsiella pneumoniae has become an urgent health care threat, causing an increasing number of high-mortality infections. Its resistance to numerous antibiotics and threat to immunocompromised patients necessitate finding new therapies to combat these infections. Previous successes in the laboratory, as well as the conservation of capsular polysaccharide (CPS) among the members of the ST258 clone, suggest that monoclonal antibody (MAb) therapy targeting the outer polysaccharide capsule of K. pneumoniae could serve as a valuable treatment alternative for afflicted patients. Here, we isolated several IgG antibodies from mice inoculated with a mixture of CR K. pneumoniae CPS conjugated to anthrax protective antigen. Two of these MAbs, 17H12 and 8F12, bind whole and oligosaccharide epitopes of the CPS of clade 2 ST258 CR K. pneumoniae, which is responsible for the most virulent CR K. pneumoniae infections in the United States. These antibodies were shown to agglutinate all clade 2 strains and were also shown to promote extracellular processes killing these bacteria, including biofilm inhibition, complement deposition, and deployment of neutrophil extracellular traps. Additionally, they promoted opsonophagocytosis and intracellular killing of CR K. pneumoniae by human-derived neutrophils and cultured murine macrophages. Finally, when mice were intratracheally infected with preopsonized clade 2 CR K. pneumoniae, these MAbs reduced bacterial dissemination to organs. Our data suggest that broadly reactive anticapsular antibodies and vaccines against clade 2 ST258 CR K. pneumoniae are possible. Such MAbs and vaccines would benefit those susceptible populations at risk of infection with this group of multidrug-resistant bacteria.

## IMPORTANCE

Carbapenem-resistant Klebsiella pneumoniae is an enteric bacterium that has been responsible for an increasing number of deadly outbreaks and hospital-acquired infections. The pathogen’s resistance to numerous antibiotics, including new drugs, leaves few therapeutic options available for infected patients, who often are too sick to fight the infection themselves. Immunotherapy utilizing monoclonal antibodies has been successful in other medical fields, and antibodies targeting the outer polysaccharide capsule of these bacteria could be a valuable treatment alternative. This study presents two anticapsular antibodies, 17H12 and 8F12, that were found to be protective against the most virulent carbapenem-resistant K. pneumoniae clinical strains. These antibodies are shown to promote the killing of these strains through several extracellular and intracellular processes and prevent the spread of infection in mice from the lungs to distal organs. Thus, they could ultimately treat or protect patients infected or at risk of infection by this multidrug-resistant bacterium.

## INTRODUCTION

The Gram-negative bacterium Klebsiella pneumoniae presents a dire health care problem. Already a common nosocomial pathogen that causes chronic urinary tract infections, pneumonias, and sepsis, the pathogen has become even more successful in the last decade because of emerging multidrug resistance to antibiotics. Infections with these strains are associated with mortality rates of >50%, particularly in hospitalized patients with comorbidities (1–3). Additionally, recent reports of carbapenem-resistant (CR) K. pneumoniae strains that have acquired a hypervirulent phenotype indicate that these strains could soon also cause disease in healthy people in the community (4). With reports of already emerging resistance to ceftazidime-avibactam (5) and limited development of other Gram-negative antibiotics (6), novel strategies to combat CR K. pneumoniae are urgently needed.

Though still an emerging field, treatment of infections with antibodies has shown some success, and to date, four such monoclonal antibodies (MAbs) are FDA licensed (7). Numerous laboratories, including ours, have demonstrated protective efficacy of anti-infective antibodies in murine models (8–12). As MAbs target specific pathogens, they are less likely to disturb the microbiome. Yet the specificity of antibodies also drives antigenic variability of pathogens and can make finding appropriate targets the most difficult hurdle in MAb development. While carbohydrate antigens, such as the antiphagocytic capsular polysaccharide (CPS), are the most accessible targets on the surface of K. pneumoniae and other members of the family *Enterobacteriaceae*, their variety has discouraged attempts to use them in immunotherapy.

In the United States, the sequence type 258 (ST258) clone comprises 80% of the CR K. pneumoniae strains and has been subcategorized into two evolutionary groups, termed clades 1 and 2 (13). Infection with clade 2 strains, which encompasses about 50% of the United States ST258 strains (1) and up to 88% of the ST258 strains in other countries, has been associated with a higher mortality rate than infection with clade 1 strains (1, 14–16). Notably, the 2009 outbreak at the National Institutes of Health Clinical Center in Bethesda, which resulted in nine deaths, was also caused by a clade 2 strain (17). Genetic differentiation of these two clades results from DNA recombination events in an ~215-kb genomic region that includes the gene cluster involved in CPS biosynthesis (13). Molecular typing of the *wzi* gene alleles, which has replaced serological typing, indicates higher conservation of CPS in clade 2 strains than in clade 1 strains, as nearly all clade 2 strains possess the *wzi-154* allele (18). We previously developed an IgM antibody, 1C9, that was shown to agglutinate 16/16 clade 2 strains tested and 1/7 clade 1 strains. However, as an IgM isotype, its affinity was too low and attempts to switch the isotype of this antibody failed (18).

In this study, we developed novel IgG antibodies by immunizing mice with protein-conjugated CPS, which improved the probability of generating IgG-producing hybridomas. We now present data showing that IgG3s 17H12 and 8F12, which bind to clade 2 CPS, exhibit broad opsonophagocytic killing efficacy against clade 2 strains and demonstrate protective efficacy in a murine intratracheal (i.t.) infection model. Interestingly, both MAbs bind to the same glycan epitope as 1C9, but both MAbs can simultaneously bind to the clade 2 CPS. In summary, we propose that 17H12 and 8F12 could provide life-saving passive immunotherapy and that the glycan epitope could constitute a potent vaccine target.

## RESULTS

### Antibody generation with clade 1 and 2 PA-conjugated CPS immunization.

Standard immunization protocols with a combination of protective antigen (PA)-conjugated CPS derived from clade 1 (strain 36) and 2 (strain 34) K. pneumoniae strains in complete Freund’s adjuvant yielded 10 different MAb-excreting hybridoma clones that bound either clade 1 or 2 CPS with high affinity. Two clones that were high producers (15 to 20 μg/ml) of anti-clade 2 MAbs were selected for further characterization (Table 1). Sequence analysis showed that the variable regions of MAbs 17H12 and 8F12 differed by only three amino acids, two of which are in complementarity-determining region 2 (CDR2), of the heavy chain V (V_H_) region. The V_H_ sequences of 8F12 and 17H12 are 98.96 and 98.61% (five and four mutated residues, respectively) identical to the IGHV5-12*02 germ line family. The MAbs have identical light chain V (V_L_) sequences, which are 98.98% (three mutations) identical to the IGKV1-135*01 germ line family. Binding affinity for purified polysaccharide was evaluated by enzyme-linked immunosorbent assay (ELISA). Both MAbs have affinities in the nanomolar range. Specifically, *K*_*d*_ binding to clade 2 CPS for 8F12 was 23.2 ± 2.4 nM and that for 17H12 was 13.2 ± 4.4 nM. In addition, 17H12 IgG3 was found to bind to clade 1 CPS with a *K*_*d*_ of 490 ± 59 nM.

**TABLE 1:** Identification of germ line variable region genes for CR CPS-specific MAbs

MAb	Isotype/clade immunization	V_H_ gene	V_H_ family	J_H_ gene	D gene	V_L_ family	V_L_ gene	J_L_ gene
8F12	IgG3/clade 2	AF305910	IGHV1-84*02	IGHJ2*01	IGHD1-3*01	AJ235936	IGKV16-104*01	IGKJ5*01
17H12	IgG3/clade 2	AF305910	IGHV1-84*02	IGHJ2*01	IGHD1-3*01	AJ235936	IGKV16-104*01	IGKJ5*01

### Both 17H12 and 8F12 bind to the hexasaccharide epitope recognized by 1C9.

First, the specificity of MAb 17H12 and 8F12 binding to CPS was tested by agglutination assays (Table 2), which demonstrated that 8F12 agglutinated 25 (96.2%) of 26 clade 2 strains and 17H12 agglutinated all 26 (100%). Next, the cross-agglutination of strains of noncognate clade 1 CPS by these MAbs was explored. 17H12 agglutinated all 16 (100%) clade 1 strains, whereas 8F12 bound to 8 (50%) of the clade 1 strains. In addition, we found that 17H12 agglutinated 11 other (non-ST258-associated) *K. pneumoniae* serotypes, including K1, K2, K3, K16, K24, K27, K35, K38, K39, K43, and K63 (data not shown).

**TABLE 2:** Agglutination of CR K. pneumoniae clinical isolates by MAbs

Clade	No. of isolates agglutinated/total (%)	
	8F12	17H12
1	8/16 (50.0)	16/16 (100)
2	25/26 (96.2)	26/26 (100)

**FIG 1:**
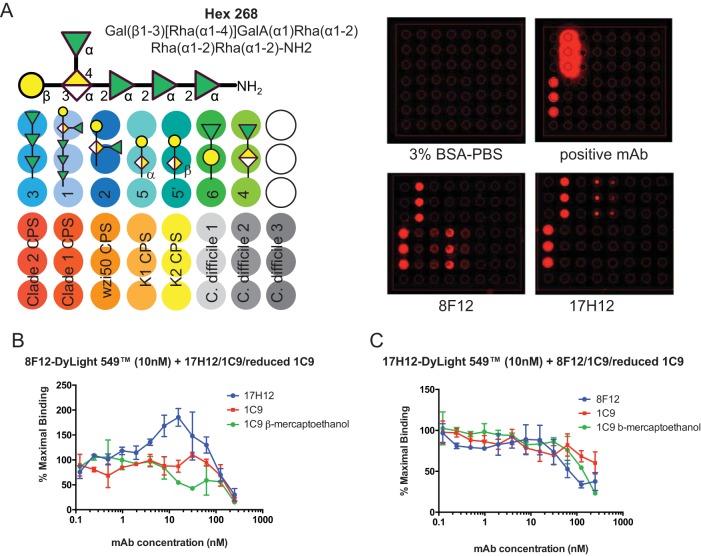
MAb binding characterization. (A) Glycan array printing schema and MAb binding. 8F12 and 17H12 bind to hexasaccharide 268 and clade 2 CPS. BSA, bovine serum albumin as negative control; and positive MAb is the previously reported 1C9 (18, 19). (B) MAbs 17H12, 1C9 (IgM), and monomeric 1C9 compete with fluorescently labeled 8F12 for binding to K. pneumoniae strain 34 CPS at higher concentrations. 17H12 increases 8F12 binding at 8F12-to-17H12 ratios of 1:0.4 to 1:7. (C) 8F12 and 1C9 compete with 17H12 for CPS binding at higher concentrations.

Next, binding was studied with recently published glycan arrays that identified a hexasaccharide epitope that is bound by 1C9, a previously described IgM that binds clade 2 CPS (Fig. 1A) (19). 1C9 was found to agglutinate fewer strains than 17H12 and 8F12, with no agglutination of clade 1 strains. We first tested if 1C9 IgM or monomeric 1C9 bind to CPS simultaneously with 17H12 or 8F12 by standard competition ELISA using fluorescently labeled 8F12 and 17H12 MAbs (Fig. 1B and C). Inhibition observed at higher concentrations in these assays could be the result of steric hindrance and/or overlapping epitopes only (20). Interestingly, 17H12 binding increased 8F12 binding to CPS, whereas 8F12 binding did not affect 17H12 binding. Next, we tested if 17H12 and 8F12 bind to previously described synthetic glycans, as well as the native CPS of K. pneumoniae strains and control CPS (Fig. 1A). Our data show that 17H12 binds to hexasaccharide 1, which was originally designed on the basis of the repeat glycan structures of the CPS isolated from the clade 2 NIH outbreak strain. Glycans 5 and 5′, which constitute shorter synthetic glycans of the same clade 2 CPS, are also recognized by this MAb. In contrast, 8F12 binds only hexasaccharide 1 and none of the shorter synthetic glycans. Instead, this MAb recognizes the K1 CPS and demonstrates weak binding to the CPS of a *wzi-50* mutant strain (clade1) and a K2 strain. These results further support the above findings, suggesting that the epitopes bound by 17H12 and 8F12 are not identical, although their variable regions differ by only two amino acids. In addition, these binding studies underscore the importance of hexasaccharide 1 as a potential vaccine target to prevent clade 2 infections.

### 17H12 and 8F12 decrease biofilm formation and serum resistance and increase complement binding.

**FIG 2:**
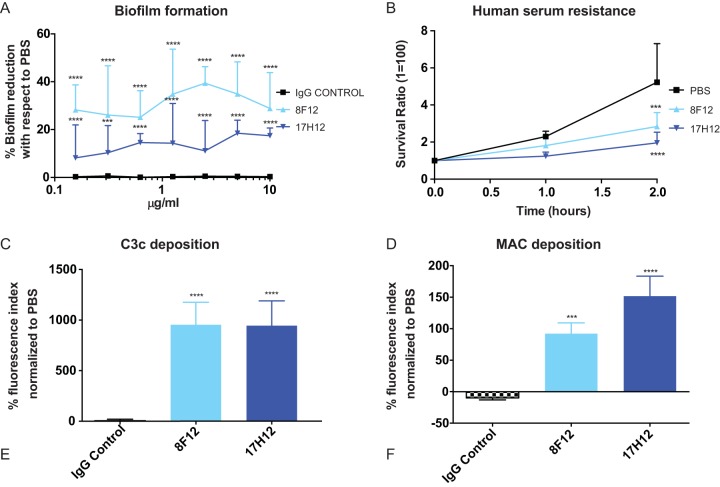
*In vitro* protection of MAbs against clade 2 CR K. pneumoniae. (A) 8F12 and 17H12 reduce biofilm formation when incorporated in cultures of CR K. pneumoniae strain 39. Log-transformed values were compared with respect to IgG control. (B) 8F12 and 17H12 prevent the replication of CR K. pneumoniae strain 39 in the presence of NHS. Survival ratio expresses the amount of growth with respect to initial inoculum. Survival ratio of ≤1 means inability to replicate, whereas survival ratios >1 expresses moderate growth in serum. PBS comparison was done with two-way ANOVA with Dunnett’s multiple comparison correction. (C) C3c complement deposition onto CR K. pneumoniae strain 39 cells is promoted in the presence of 8F12 and 17H12. (D) MAC deposition onto CR K. pneumoniae strain 39 cells is promoted by 8F12 and 17H12. Log-transformed values from C and D were compared with the IgG control. All experiments were carried out independently in triplicate. *P* values were determined by one-way ANOVA with multiple-comparison correction. *, *P* < 0.05; **, *P* < 0.01; ***, *P* < 0.001; ****, *P* < 0.0001.

The formation of CR K. pneumoniae strain 39 biofilms on polystyrene plates was measured after 16 h of growth in the presence or absence of MAbs. 8F12 significantly decreased biofilm formation with respect to IgG control even at concentrations as low as 0.156 mg/ml (*P* < 0.01) (Fig. 2A). A similar, albeit less pronounced, effect was observed with 17H12, whereas the IgG isotype control MAb had no effect in these assays. The polysaccharide capsule also shields K. pneumoniae from the bactericidal effect of serum, which enables it to grow in blood (3). Our data show that coincubation of CR K. pneumoniae strain 39 with either 8F12 or 17H12 significantly impairs the ability of the strain to replicate in 75% normal human serum (Fig. 2B). Consistent with that observation, both MAbs increased C3c complement deposition on CR K. pneumoniae strain 39 (Fig. 2C, P < 0.001). Furthermore, the deposition of the membrane attack complex (MAC) was also found to be significantly increased (Fig. 2D, P < 0.01 for 8F12 and *P* < 0.001 for 17H12). Neither the deposition of C3c complement nor the deposition of MAC components was found to be increased when CR K. pneumoniae was coincubated with an IgG control antibody.

### Antibodies promote intra- and extracellular killing of CR K. pneumoniae.

**FIG 3:**
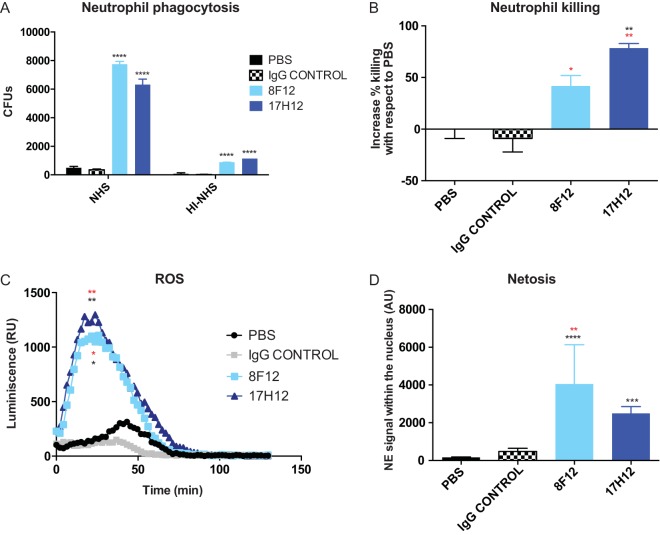
Clade 2 MAbs promote neutrophil phagocytosis and killing of clade 2 CR K. pneumoniae. (A) Neutrophil phagocytosis of CR K. pneumoniae strain 39 cells was increased 2 and 3 h after incubation with 8F12 and 17H12 as detected by pHrodo signaling. (B) Increased neutrophil killing of CR K. pneumoniae strain 39 cells in the presence of 8F12 and 17H12. (C) Incubation with specific MAbs increased ROS production. (D) MAbs increased NETosis production. Black asterisks mean comparison with respect to PBS, whereas red asterisks mean comparison with respect to IgG control. *P* values were determined by one- or two-way ANOVA with multiple-comparison correction. *, *P* < 0.05; **, *P* < 0.01; ***, *P* < 0.001; ****, *P* < 0.0001. RU, relative units; AU, arbitrary units.

Opsonophagocytic killing assays can predict the protective efficacy of anticapsular MAbs. Therefore, the phagocytosis of clade 2 CR K. pneumoniae strains by primary human neutrophils was evaluated. Clade 2 CR K. pneumoniae strain 39 was poorly phagocytosed by neutrophils in the presence of phosphate-buffered saline (PBS) or IgG control antibody, whereas coincubation with 8F12 or 17H12 significantly increased the phagocytosis of CR K. pneumoniae strain 39 even in the absence of active normal human serum (*P* < 0.05 and *P* < 0.0001, respectively, Fig. 3A). Killing was also significantly enhanced by 8F12 and even more efficiently by 17H12 (*P* < 0.05 and *P* < 0.0001, respectively, Fig. 3D). Again, these effects were found to be dependent on a CR K. pneumoniae-specific antibody. Most importantly, the opsonophagocytic killing efficacy of 17H12 was confirmed in 19 other clinical clade 2 CR K. pneumoniae strains (Fig. S1), emphasizing the potential therapeutic use of anticapsular antibodies despite CPS variability.

10.1128/mBio.00091-18.1 FIG S1  J744.16 phagocytosis of CR K. pneumoniae clade 2 strains is enhanced by incubation with 17H12. Download FIG S1, PDF file, 0.4 MB. Copyright © 2018 Diago-Navarro et al. 2018 Diago-Navarro et al. This content is distributed under the terms of the Creative Commons Attribution 4.0 International license.

Reactive oxygen species (ROS) production is important for neutrophil-mediated killing and was examined in the presence of MAbs. CR K. pneumoniae clade 2 strain 39 cocultured with neutrophils poorly induced ROS production alone, but its induction of ROS significantly increased in the presence of 8F12 or 17H12 (area-under-the-curve analysis, *P* < 0.05 and *P* < 0.01, respectively, Fig. 3C). NETosis is a mechanism by which neutrophils excrete neutrophil extracellular traps (NETs) that trap bacteria and promote extracellular killing by minimizing damage to host cells. This mechanism of extracellular killing can be enhanced by MAbs (8). We evaluated the mobilization of neutrophil elastase (NE) to the nucleus, an established NETosis marker (21). We found that both 8F12 and 17H12 promoted the colocalization of NE in the nucleus, whereas PBS or IgG control treatment (Fig. 3D) had no effect.

### 17H12 and 8F12 promote *in vivo* protection in a murine i.t. infection model.

**FIG 4:**
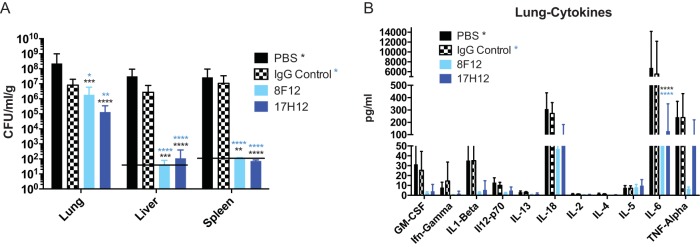
17H12 promotes *in vivo* protection in an i.t. murine infection model. (A) Organ bacterial burdens were decreased in the lungs, liver, and spleen after 17H12 or 8F12 treatment. (B) The lung cytokine IL-6 level was significantly reduced. *P* values were determined by two-way ANOVA with multiple-comparison correction. *, *P* < 0.05; **, *P* < 0.01; ***, *P* < 0.001; ****, *P* < 0.0001. Black asterisks denote *P* values compared to PBS, and blue asterisks denote *P* values compared to an isotype IgG control.

Although clade 2 strains constitute the more virulent CR K. pneumoniae clade in human infection, murine infection usually does not result in death unless very large inocula are used. Therefore, we investigated *in vivo* protection of mice by 17H12 and 8F12 by quantifying tissue bacterial burdens in a pulmonary infection model. These experiments document a significant decrease in organ CR K. pneumoniae strain 39 burdens when mice were coinjected with 17H12 or 8F12, whereas no effect on the organ bacterial burden was observed in PBS- or isotype control antibody (Fig. 4A)-treated mice. In the liver and spleen, bacterial counts were found to be under the limit of detection in MAb-treated mice, either because dissemination to the specific organ was prevented or because clearance of the organ was promoted. Consistent with the observed protective efficacy, *in vivo* cytokine analysis of infected tissue also demonstrated differences (Fig. 4B). Most notably, levels of the proinflammatory mediator interleukin-6 (IL-6), which enhances neutrophil-mediated killing, was significantly reduced in the presence of both 8F12 and 17H12 and levels of granulocyte-macrophage colony-stimulating factor, gamma interferon, IL-1β, and IL-18 were also decreased. Histological analysis of lung sections confirmed less recruitment of inflammatory cells in MAb-treated mice than in controls (data not shown).

## DISCUSSION

CR K. pneumoniae strains continue to be the leading multidrug-resistant members of the family *Enterobacteriaceae*, and specifically infections with clade 2 ST258 CR K. pneumoniae strains are associated with poor clinical outcomes (1, 5). Given the limited new antibiotic treatments in development, new therapies and preventive measures are crucial to limit the health care burden these infections cause. Ample animal data have demonstrated protective efficacy of vaccines and MAbs targeting the polysaccharide capsule of various microbes (22–24). Vaccination with protective epitopes of CR K. pneumoniae CPS could prevent infections and colonization in individuals in high-risk settings such as long-term hospitalization or nursing home residency. Meanwhile, MAbs could be used to treat patients who cannot develop an effective immune response. Though our MAbs might treat only infections by clade 2 CR K. pneumoniae strains, the Prevnar 7 antipneumococcal conjugate vaccine is protective, despite covering only 50% of the serotypes prevalent in some countries (25, 26). Also, protection against 50% of the CR K. pneumoniae strains in the United States and 80% of those in other countries could have a notable impact on the outcome of these infections because they are more likely to lead to death (1).

Our data indicate that 8F12 and 17H12 recognize similar antigen structures of the clade 2 CPS, as both bind to hexasaccharide 1, the epitope that 1C9 also recognizes (19). However, certain differences were found. Specifically, only 17H12 is able to bind to the smaller oligosaccharides, whereas neither 8F12 nor 1C9 is (this work and reference 19). Interestingly, the 8F12 and 17H12 sequences differ by only two amino acids in V_H_ CDR2 (56Gly 57Ser and 56Ser 57Asn, respectively). Single amino acid changes in CDR2 have been sufficient to increase MAb epitope binding elsewhere (27) and could facilitate 17H12 binding to smaller oligosaccharides. Interestingly, biofilm formation was inhibited more in the presence of 8F12 than in the presence of 17H12, further supporting the notion that the interactions of the MAbs with the CPS differ. Also of note is the enhancement of 8F12 binding to CR K. pneumoniae CPS upon binding by 17H12, which could indicate cooperative antigen binding (28). Such cooperativity is responsible for transforming nonneutralizing ebolavirus antibodies into potent neutralizers (29) and also for synergistic effects observed when MAbs are combined for treatment (30), which could also be considered for 17H12 and 8F12. Although it is still unclear, it is possible that binding of 17H12 exposes an epitope that can now better interact with the variable region of 8F12. Alternatively, such binding cooperativity has also been proposed to occur through non-covalent Fc-Fc intermolecular interactions between two mouse IgG3 antibodies (43). Crystallographic investigations could be employed to test these hypotheses.

ST258 strains have been shown to have high resistance to complement-mediated killing and phagocytosis by neutrophils (3, 31, 32). Both 17H12 and 8F12 efficiently promote the deposition of complement and MAC and, as a result, reduce resistance to human serum. In addition, neutrophil phagocytosis is significantly increased and induces ROS and effective killing by neutrophils. It also increases extracellular killing by NETosis, another mechanism to facilitate the clearance of K. pneumoniae infections (33). This effect has been pointed out as a potential therapeutic approach to the treatment of CR K. pneumoniae infections (32).

The lack of good *in vivo* animal models that mimic the natural pathogenesis of CR K. pneumoniae infections, which are characterized by a prolonged smoldering course and occasionally even sustained bacteremia (34), impedes the development of alternative therapeutic modalities that do not target lipopolysaccharide. Even clade 2 CR K. pneumoniae strains are not very virulent in murine models unless large inocula (>10^8^ bacteria) are used, and in these cases, mice typically die of septic shock in <48 h (3, 18, 31, 35). In our experience, even a 0.5-log reduction of bacterial inocula prevents mouse lethality (data not shown). Furthermore, this time course and modality of death do not align with the median time to death seen in CR K. pneumoniae-infected patients, which is more than a week after evidence of bacteremia (1). Similar to studies with Mycobacterium tuberculosis (36), protective efficacy of MAbs was demonstrated when preincubated CR K. pneumoniae inocula were injected i.t. Both MAbs were able to decrease lung, liver, and spleen bacterial burdens effectively, and they reduce proinflammatory cytokines. Organ bacterial burden reduction and cytokine analysis are normally used as indications of MAb efficacy (8, 11, 37, 38). The dual activity of IL-6, a pro- and anti-inflammatory cytokine, has been found to be associated with severity of disease and systemic inflammatory responses in infections by other pathogens such as influenza virus and Streptococcus pneumoniae. There, protection by MAb has been shown to decrease IL-6 levels and improve survival (38, 39).

In summary, these two MAbs can be considered candidates for an antibody-based approach to the treatment of CR K. pneumoniae-infected patients otherwise with limited therapeutic options. Furthermore, glycan array binding studies highlight the importance of previously described hexasaccharide 1, which constitutes a relevant epitope on all of the clade 2 strains tested. This oligosaccharide should be further explored for vaccine development.

## MATERIALS AND METHODS

### CR K. pneumoniae strains.

CR K. pneumoniae strains were collected from patients at the Montefiore Medical Center (MMC) and the Stony Brook University (SBU) hospital under institutional review board (IRB)-approved protocols. Clade 2 MMC K. pneumoniae strains 34, 39, M1, M5, M6, M25, M26, M48, M49, M47, and M13 and clade 1 MMC K. pneumoniae strain 36 have been described previously (18). Clade 2 SBU strains 4, 12, 20, 32, 34, 35, 36, 45, 86, and 208 were isolated at the SBU hospital. K. pneumoniae strains were cultured in Luria-Bertani (LB) broth or agar at 37°C.

### CPS purification and conjugation.

CPS was isolated and conjugated to *Bacillus anthracis* PA by the 1-cyano-4-dimethylaminopyridinium tetrafluoroborate method as previously described (8).

### MAb production.

MAbs to ST258 CPS were generated by immunizing 6- to 8-week-old BALB/c mice with 100 μg of PA-conjugated CPS of K. pneumoniae strains 36 and 34 in complete Freund’s adjuvant, followed by boosters of PA-conjugated CPS in incomplete Freund’s adjuvant 2, 4, and 6 weeks later. Fusion and cloning were performed as previously described (8). The 8F12 and 17H12 variable regions were sequenced by GenScript and analyzed with the International ImMunoGeneTics Information System software program. The affinities of the MAbs were calculated by ELISA as previously described (8). Labelling of MAbs with DyLight 549 (ThermoFisher Scientific) was done following manufacturer’s protocol. Reduction of the multimeric form of IgM 1C9 MAb was done with 0.15 M of β-mercaptoethanol in TBS (1:1 v/v) for 1 h at 37°C as previously reported (44). Either commercial murine IgG3 (Crown Biosciences Inc.), which recognizes hen egg lysozyme, or 9D8, an IgG3 MAb that binds to arabinomannan (36), was used as an IgG control antibody.

### Agglutination assays.

Agglutination assays were carried out as previously described (40).

### Glycan arrays.

Development of glycan arrays and their use in binding assays are described elsewhere (19).

### Biofilm production assays.

Biofilm production assays were carried out as previously described after 16 h of growth (18).

### Complement deposition assays.

Testing for C3c complement deposition was performed as previously described (8). Assays querying MAC deposition were performed similarly but with 1 h of incubation of normal human serum (NHS) plus bacteria with or without MAbs. MAC was detected with an Alexa Fluor 647-conjugated anti-human C5b-9 (Bioss Antibodies) with 20 min of incubation at 4°C. Flow cytometry was used to quantify the complement deposition signal. Quantification was performed by multiplying the percentage of bacteria moving into the gate by the average fluorescence of a defined population (X-mean), to give a fluorescence index. Results of both assays are expressed by the increment of fluorescein isothiocyanate or Alexa Fluor 647 signal with respect to PBS incubation.

### Human serum resistance assays.

Human serum resistance assays were performed as previously described (8).

### NETosis.

NETosis was carried out as previously described (41). Colocalization of NE was quantitated with ImageJ. Ten different cells were analyzed per condition.

### Phagocytosis experiments.

J744.16 murine macrophages and human primary neutrophils were used for phagocytosis assays with bacteria labeled with pHrodo (Life Technologies, Inc.) as previously described (8). Neutrophils were purified as previously described (8). Neutrophil phagocytosis was carried out at a MOI of 0.1. Cells (1 × 10^6^/well) were seeded and 10^5^ CR K. pneumoniae cells incubated for 1 h with PBS, and IgG control or 17H12 were added to the wells. After 1 h, extracellular bacteria were washed out with fresh media twice and incubated for 30 min with 100 μg/ml of polymyxin B. Then, polymyxin B was washed out with fresh media twice and macrophage cells were lysed with H_2_O. Bacteria were plated in LB plates in triplicates with the appropriate dilutions. Neutrophil killing was assayed at a multiplicity of infection (MOI) of 0.1 as previously described (41). *In vitro* murine J744.16 cell line phagocytosis was assayed by plating 5 × 10^5^ cells/well. After overnight culture, 10^6^ cells of different CR K. pneumoniae strains were incubated for 1 h with PBS, IgG control, or 17H12 and added to the wells at an MOI of 1:1. After 30 min of incubation, extracellular bacteria were washed out with fresh medium twice and incubated for 30 min with 100 μg/ml polymyxin B. The polymyxin B was then washed out with fresh medium twice and macrophages were lysed with H_2_O. Bacteria were plated on LB plates in triplicate with the appropriate dilutions.

### Neutrophil ROS production.

ROS production was assayed with luminol as previously described, with modifications (42). Briefly, 5 × 10^5^ neutrophils in RPMI medium plus 10% fetal bovine serum were plated on a 96-well luminometer plate (Nunc, Roskilde, Denmark). A total of 5 × 10^6^ CFU of CR K. pneumoniae previously incubated for 1 h with 40 μg/ml PBS, IgG control, or specific MAbs were added with luminol. Immediately after stimulus addition, 50 μl of RPMI medium containing 200 μM luminol was distributed into each well. ROS production was determined by measuring luminescence every 2 min for 2 h at 37°C in a microplate reader (Molecular Devices). Data are expressed as relative luciferase units or total relative luciferase units under the curve in 90 min. Statistical significance of differences was calculated by one-way analysis of variance (ANOVA) from data of three independent experiments.

### I.t. infection model.

Female BALB/c mice 6 to 8 weeks old were used. CR K. pneumoniae strain 39 (2 × 10^8^ CFU/ml) was incubated with 17H12, IgG control, or PBS at 5 mg/ml for 1 h. A 50-μl volume with 10^7^ CFU preincubated with 250 μg of MAbs was then injected i.t. After 24 h, mice were euthanized and the livers, spleens, and lungs of eight mice were processed for enumeration of bacteria in homogenized tissue, histology observation, and cytokine analysis.

### Statistical analysis.

Statistical tests were performed with GraphPad Prism 6 for Mac. Data for biofilm, complement deposition, netosis, and neutrophil phagocytosis were log-transformed and tested for normality and equality of variance and were deemed to be parametric. For multigroup comparisons of parametric data (e.g., phagocytosis, log-transformed CFU counts, log-transformed complement deposition), ANOVA and *post hoc* analysis with Tukey’s, Šidák’s, or Dunnett’s comparison test were used. Data in figures are deposited as raw data, not log-transformed.

### Ethics statement.

Animal study protocols were approved by the Animal Committee (IACUC) at SBU (approval no. 628253). This study is in strict accordance with federal, state, local, and institutional guidelines that include the Guide for the Care and Use of Laboratory Animals, the Animal Welfare Act, and Public Health Service Policy on Human Care and Use of Laboratory Animals. All surgery was performed under ketamine-and-xylazine anesthesia, and every effort was made to minimize suffering. Human studies were approved by the SBU Human Subjects Committee (IRB approval no. 718744). Healthy donors gave written informed consent for blood donation.
