# Erratum for Diago-Navarro et al., “Novel, Broadly Reactive Anticapsular Antibodies against Carbapenem-Resistant Klebsiella pneumoniae Protect from Infection”

**DOI:** 10.1128/mBio.01005-18

**Published:** 2018-06-12

**Authors:** Elizabeth Diago-Navarro, Michael P. Motley, Gonzalo Ruiz-Peréz, Winnie Yu, Julianne Austin, Bruna M. S. Seco, Guozhi Xiao, Aniska Chikhalya, Peter H. Seeberger, Bettina C. Fries

**Affiliations:** a Department of Medicine, Infectious Disease Division, Stony Brook University, Stony Brook, New York, USA; b Department of Molecular Genetics and Microbiology, Stony Brook University, Stony Brook, New York, USA; c Universidad Francisco de Vitoria, Pozuelo de Alarcón, Madrid, Spain; d Department of Biomolecular Systems, Max Planck Institute of Colloids and Interfaces, Potsdam, Germany

## ERRATUM

Volume 9, issue 2, e00091-18, 2018, https://doi.org/10.1128/mBio.00091-18. After careful review of the legend of Fig. 3, we realized that there was an error in the section describing panel A. The revised legend for Fig. 3A should read “Neutrophil phagocytosis of CR K. pneumoniae strain 39 cells was increased after incubation with 8F12 and 17H12 in the presence of NHS or HI-NHS.” We also noticed that on PDF page 5 (first paragraph), the statement about neutrophil killing should reference Fig. 3B, not Fig. 3D.

