# Supplementary material for: Novel, Broadly Reactive Anticapsular Antibodies against Carbapenem-Resistant Klebsiella pneumoniae Protect from Infection
Source: mBio. 2018 Apr 3;9(2):e00091-18. doi: 10.1128/mBio.00091-18 (PMC5885035; doi:10.1128/mBio.00091-18)
Supplement: FIG S1 [file mbo002183829sf1.pdf]

A

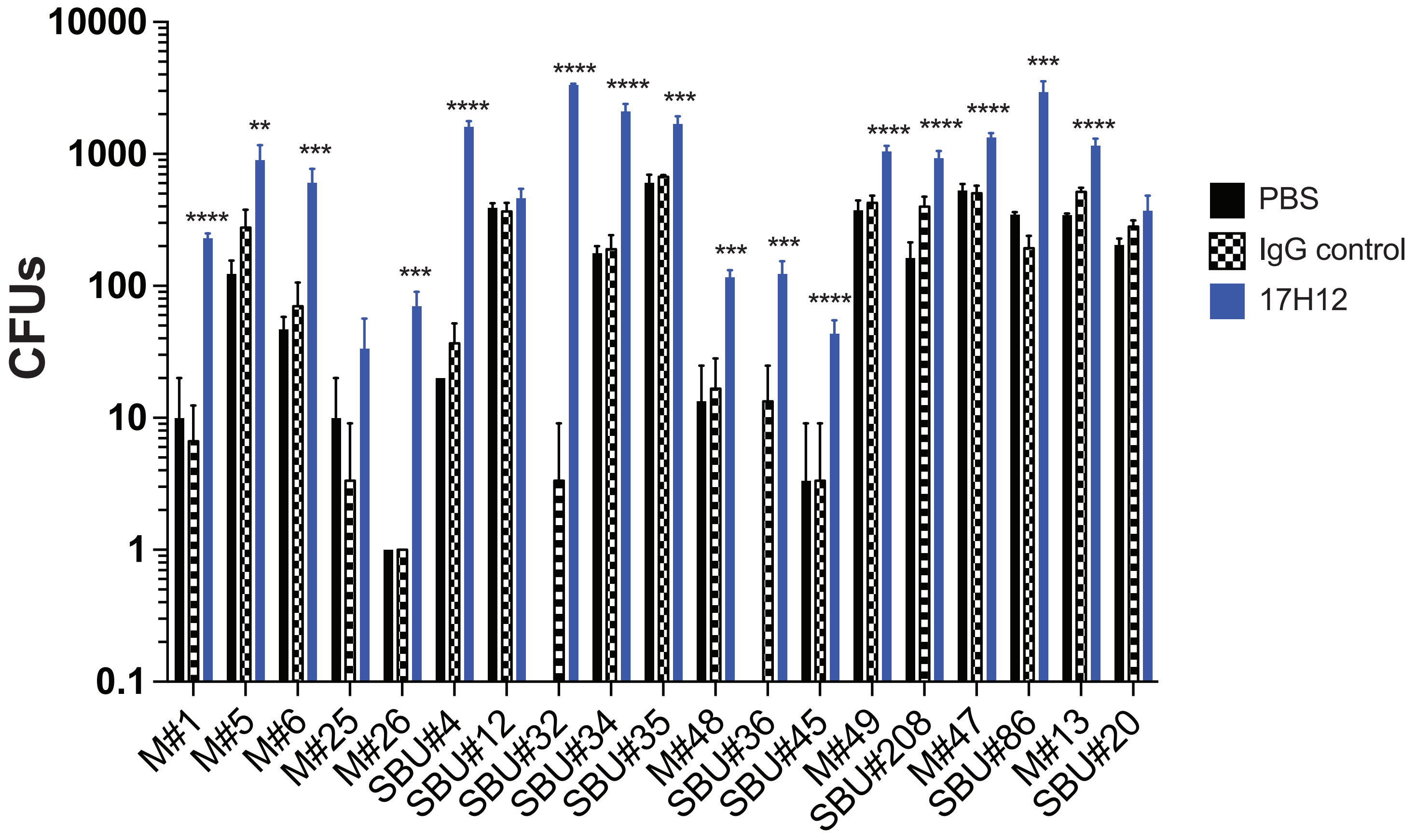

Supplementary Figure 1: J744.16 phagocytosis of CR-Kp clade 2 strains is enhanced by the incubation with 17H12. P-values were determined by one-way ANOVA and are depited all together for visualization purposes. ** denotes p-value <0.01, *** denotes p-value <0.001 and **** denotes p-value <0.0001.
